# From Bulk to Binding: Decoding the Entry of PET into Hydrolase Binding Pockets

**DOI:** 10.1021/jacsau.4c00718

**Published:** 2024-09-26

**Authors:** Anna Jäckering, Frederike Göttsch, Moritz Schäffler, Mark Doerr, Uwe T. Bornscheuer, Ren Wei, Birgit Strodel

**Affiliations:** † Institute of Theoretical and Computational Chemistry, Heinrich Heine University, Düsseldorf, Universitätsstr. 1, 40225 Düsseldorf, Germany; ‡ Institute of Biological Information Processing: Structural Biochemistry (IBI-7), Forschungszentrum Jülich, Wilhelm-Johnen-Straße, 52428 Jülich, Germany; § Department of Biotechnology & Enzyme Catalysis, Institute of Biochemistry, University of Greifswald, Felix-Hausdorff-Str. 4, 17487 Greifswald, Germany

**Keywords:** poly(ethylene terephthalate) (PET), PET hydrolases, PET binding, molecular dynamics (MD), replica exchange MD, binding modes, enzyme engineering, kinetics

## Abstract

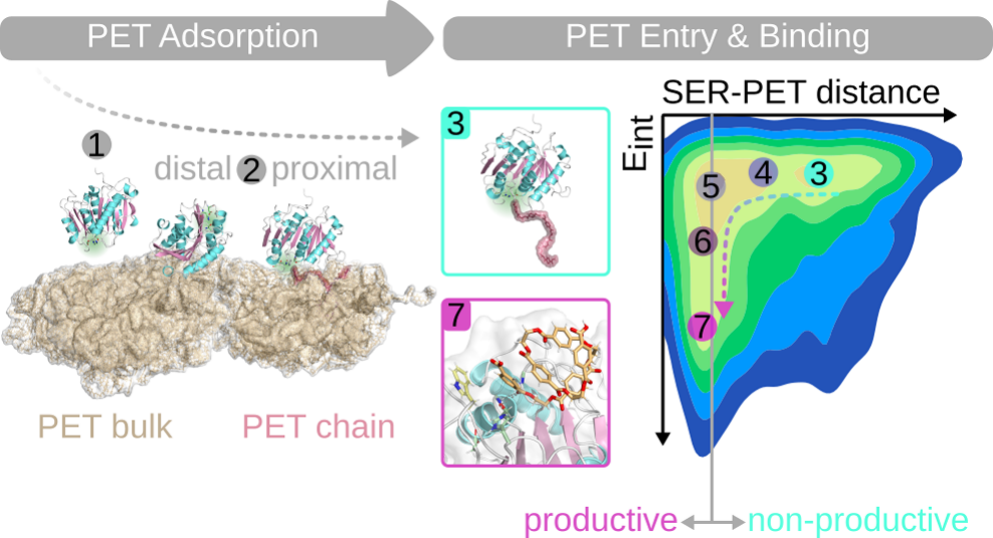

Plastic-degrading enzymes facilitate the biocatalytic recycling of poly(ethylene terephthalate) (PET), a significant synthetic polymer, and substantial progress has been made in utilizing PET hydrolases for industrial applications. To fully exploit the potential of these enzymes, a deeper mechanistic understanding followed by targeted protein engineering is essential. Through advanced molecular dynamics simulations and free energy analysis methods, we elucidated the complete pathway from the initial binding of two PET hydrolases—the thermophilic leaf-branch compost cutinase (LCC) and polyester hydrolase 1 (PES-H1)—to an amorphous PET substrate, ultimately leading to a PET chain entering the active site in a hydrolyzable conformation. Our findings indicate that initial PET binding is nonspecific and driven by polar and hydrophobic interactions. We demonstrate that the subsequent entry of PET into the active site can occur via one of three key pathways, identifying barriers related to both PET–PET and PET–enzyme interactions, as well as specific residues highlighted through *in silico* and *in vitro* mutagenesis. These insights not only enhance our understanding of the mechanisms underlying PET degradation and facilitate the development of targeted enzyme enhancement strategies but also provide a novel framework applicable to enzyme studies across various disciplines.

## 1 Introduction

The escalating plastic consumption leading to a rise in plastic waste highlights the need for efficient and environmentally friendly recycling processes.^1^ Enzymatic depolymerization of plastics, notably poly(ethylene terephthalate) (PET), has emerged as a promising approach for monomer recovery, aiming to address the challenges of current recycling methodologies.^2−6^ PET is an aromatic, semicrystalline thermoplastic that can be enzymatically degraded into key building blocks including 2-hydroxyethylterephthalate, which can then be further hydrolyzed into its monomers terephthalate and ethylene glycol by the same or different enzymes.^4,7−9^ In 2005, enzymatic degradation of low-crystallinity PET has been reported for the first time with a *Thermobifida fusca* hydrolase.^10^ A milestone in microbial PET degradation was achieved 11 years later with the discovery of *Ideonella sakaiensis*, which features a two-enzyme system for the depolymerization of PET and its degradation products as the main source of carbon and energy for the bacterial growth.^11^ However, the activity and thermostability of wild-type (WT) PET hydrolases are often insufficient for their industrial application. To this end, diverse PET hydrolases based on the leaf-branch-compost cutinase (LCC) and the polyester hydrolase 1 (PES-H1) have been recently engineered, yielding two of the currently most active and thermostable PET hydrolases, the LCC F243I/D238C/S283C/Y127G (ICCG) variant^3^ and the PES-H1 L92F/Q94Y (FY) variant.^12^ The development of LCC^ICCG^ by the French company Carbios enabled the construction of the first industrial plant for enzymatic PET recycling (capacity of 50,000 tons/year), which is due to go into operation shortly.^3,13,14^ Nonetheless, challenges remain for optimizing enzymatic kinetics of the PET hydrolases.^3,4,15^

To make progress here, a deeper understanding of the entire PET degradation process is required. This process consists of initial adsorption of the enzyme to the PET material, followed by a single PET chain from this bulk entering the active site of the PET hydrolase and assuming a binding position productive for subsequent hydrolysis. It was suggested that the PET hydrolases adsorb nonspecifically on the surface of the PET bulk, resulting in either a productive complex that enables PET degradation by binding close to the active site, or in an unproductive complex that cannot hydrolyze PET.^16,17^ To enhance the activity of PET hydrolases, efforts have focused on improving PET adsorption by modifying surface properties through charge, polarity, and hydrophobicity adjustments.^18^ Increased PET adsorption was observed after the introduction of positive charges^19,20^ and increased hydrophobicity of the enzyme surface.^21−24^ According to the Sabatier principle, optimal catalysis occurs when interactions between catalyst and substrate are of intermediary strength. For LCC it was demonstrated that its activity increased when its substrate affinity is weakened through the addition of a cationic surfactant.^25^ However, achieving the optimal balance between binding affinity and activity remains a challenge, necessitating further exploration of the initial PET adsorption process and the transition to a productive PET binding mode at the active site.

Design strategies for PET hydrolases often target the catalytic binding cleft to bolster enzymatic activity, often inspired by the amino acid compositions of different hydrolases. This approach resulted in the PES-H1^FY^ variant, which was motivated by the thermostable DuraPETase.^12,26^ Computational methods offer insights into the importance of specific residues in the binding site, which complement crystal structures of PET hydrolase with small ligands reported recently.^9,12,19,27−31^ Based on the analysis of these structural complexes, it was hypothesized that widening the binding cleft would increase the accessibility of the active site and consequently the enzyme’s activity,^32−35^ while narrowing the binding site could enable specific interactions with PET that promote substrate binding and enzyme activity.^36^ Introducing aromatic, hydrophobic, or hydrogen bond-forming residues into PET hydrolases has been observed to enhance their efficiency.^33,35,37,38^ Particularly noteworthy is a highly conserved tryptophan near the binding cleft, which appears to be crucial for PET degradation and whose flexibility varies between mesophilic and thermophilic enzymes due to neighboring amino acids, affecting its functional “wobbling” ability.^31,39−41^ However, not all findings align on the factors crucial for productive PET binding, underscoring the need for further analysis to pinpoint consistent indicators of successful binding for subsequent PET degradation. While past computational studies mainly utilized docking^3,31,32,42,43^ in combination with short molecular dynamics (MD) simulations,^12,36,44−47^ these methods might inadequately capture the intricacies of the binding process, specifically the dynamic interplay between the binding site and PET during PET entry into the active site. However, these dynamic processes have been suggested to be important factors for PET complexation and thus degradation.^4,44,45,48−50^

To fill this knowledge gap, we performed thorough computational simulations in combination with experimental studies on two metagenome-derived thermophilic PET hydrolases, LCC (catalytic triad: S165, D210, H242) and PES-H1 (catalytic triad: S130, D176, H208). We subjected both enzymes to extensive MD as well as Hamiltonian replica exchange MD (HREMD) simulations to elucidate their adsorption to a PET bulk, tracking PET entry into the active site until PET binds in a productive mode to allow for hydrolysis. PET entry is analyzed using a free energy surface-based approach to reveal residues that affect the energy barrier during the entry process, whereupon residue substitutions are predicted and then evaluated *in vitro*. Our approach is the first to show how PET reaches the active site, uncovering important information about the dynamics of both the enzyme and PET, as well as the interactions between polymer and enzyme.

## 2 Results and Discussion

**Figure 1:**
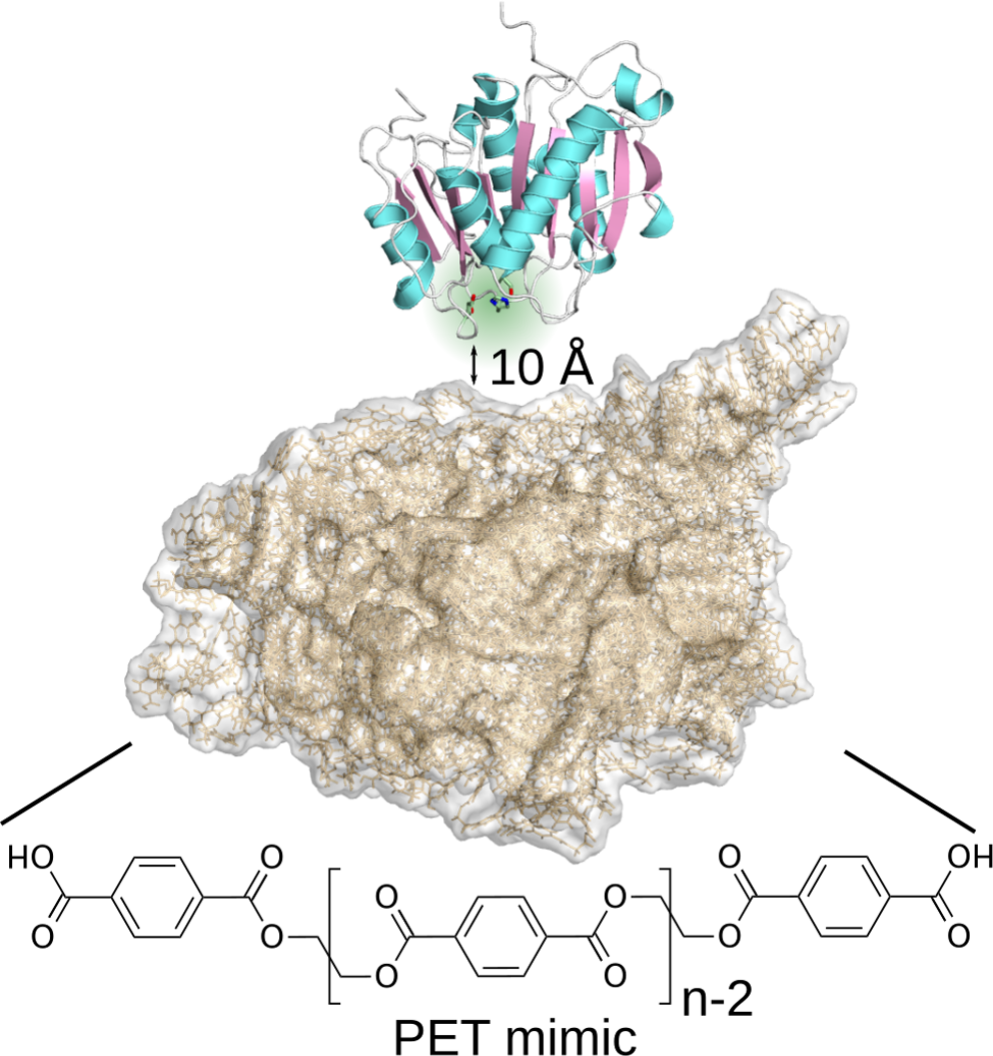
Representative starting structure for the MD simulations of enzyme adsorption on amorphous PET (without water and ions being shown). The system comprises one PET hydrolase, which is shown as cartoon and with the catalytic triad highlighted in green. The enzyme was placed with random orientation at least 10 Å away from the PET bulk, which consists of 100 PET-9mers and is shown as brown surface.

To explore the complete enzyme binding process from LCC and PES-H1 interacting with a PET bulk to a PET chain positioned in the active site for hydrolysis, our study commenced by randomly orienting both LCC and PES-H1 at a minimum distance of 10 Å from an amorphous PET bulk (Figure 1) and conducting a total of 10.5 μs of MD simulations for each enzyme. In these simulations, we observed enzyme adsorption on the PET surface at sites proximal and distal to the binding cleft, but the PET failed to enter into the active site. Therefore, we continued the simulations by applying the enhanced-sampling HREMD method (accumulating further 22.0 and 21.4 μs of sampling of LCC and PES-H1, respectively), where we used only one single PET chain closest to the binding cleft allowing PET to enter the active site and adopt productive binding poses. The results of these two sets of simulations are discussed in detail below along with the wet lab studies to consolidate our *in silico* findings. Detailed information on all simulations and experiments can be found in the Supporting Information (SI), including the workflow of the study depicted in Figure S1.

### 2.1 PET Adsorption to the Surface of the Enzyme

#### 2.1.1 Both Enzymes Readily Adsorb to PET, But without a Strong Preference for the Binding Cleft

**Figure 2:**
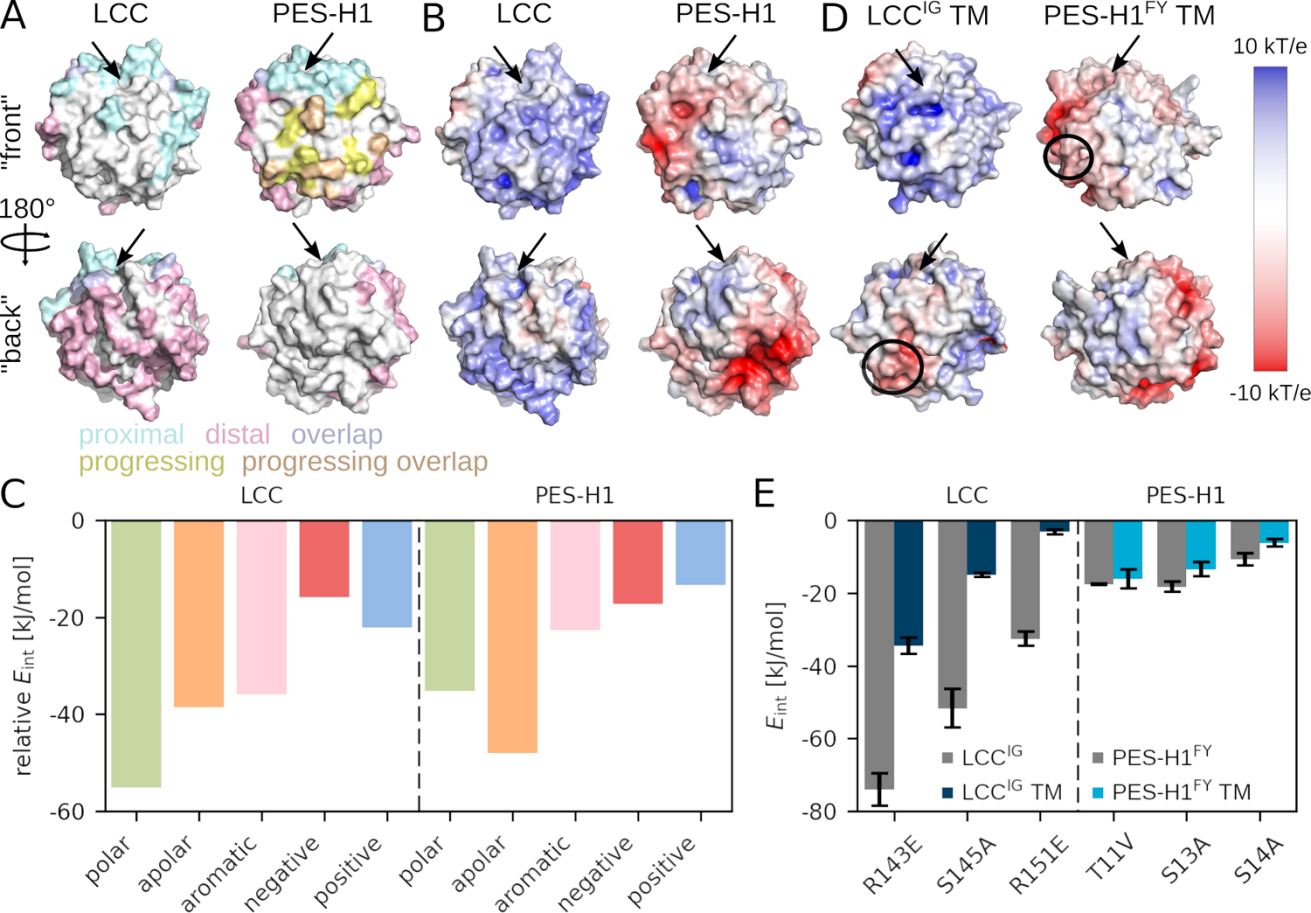
Analysis of LCC and PES-H1 adsorption on amorphous PET. (A) On the enzyme surfaces (LCC, left; PES-H1, right), the residues that are in contact with PET for at least 75% of the simulation time (using all six simulations per enzyme) are highlighted (distance limit 3 Å). Contacts are differentiated into proximal (blue) and distal (rose) sites with respect to the catalytic site (indicated by an arrow), with overlapping proximal/distal regions colored purple. One of the PES-H1 simulations revealed a relocation of the enzyme on the PET bulk, with the transition from distal to proximal binding highlighted in yellow (overlaps with previously sampled contacts are shown in brown). (B) EPS for LCC (left, net charge: +5) and PES-H1 (right, net charge: −5), according to the color scale on the right. (C) PET interaction energies *E*_int_ with physicochemically similar residues in LCC and PES-H1 are shown. The energies are scaled relative to the number of corresponding residues in contact with PET and averaged over all simulations. A detailed version of this graph, showing the energetic contributions by the 20 amino acids and with standard error of the mean is shown in Figure S2. (D) EPS of the LCC^IG^ triple mutant (TM) R143E/S145A/R151E and the PES-H1^FY^ TM T11 V/S13A/S14A. The locations of the mutations are denoted by black circles and the catalytic site is indicated by an arrow. (E) The interaction energies between PET and the residues at the mutation sites are shown for the parent enzymes LCC^IG^ and PES-H1^FY^ (gray) and the corresponding TM (blue).

Adsorption to the PET bulk was sampled in all six of the 1.5 μs simulations for both LCC and PES-H1. In addition, the initial enzyme–PET interactions of considerable strength with energies below −10 kcal·mol^–1^ were established early in the simulations (PES-H1: 2.0–40.2 ns; LCC: 0.8–15.0 ns) and abundant contacts (defined based on the residue–PET distance requirement ≤3 Å) were observed at the surfaces of LCC and PES-H1, suggesting that initial binding is based on general features such as charge and polarity rather than specific recognition sites.^51−53^ These contacts, which are present on the entire surface of both enzymes, were divided into proximal and distal binding based on the proximity of PET to the catalytic serine within 12 Å; they are shown in blue and rose, respectively, in Figure 2A. To follow the progression of the contacts during the simulations, we compared the contact areas across the simulation duration. Minimal enzyme reorientations occurred on the PET surface, with the majority of contacts remaining stable for 75% or more of the total simulation time. In the case of proximal binding at the end of the simulations, we extended the corresponding simulations to 2 μs to see if further PET progression toward the active site would occur, which was mostly not the case. When the initial contacts were made in proximal regions, the PET contact area remained close to the active site (LCC: 4 out of 6; PES-H1: 2 out of 6 simulations), while in the cases where the initial contacts were located in distal regions, only a negligible approach to the active site was observed. Only in one of the PES-H1 simulations, proximal contacts could be established starting from distal ones; this contact progression is marked in yellow in Figure 2A. To gain insights into the driving forces behind initial enzyme–PET binding and to potentially enhance proximal binding through protein engineering, we analyzed the physicochemical nature of the contacts between the enzymes and the PET bulk. This analysis also accounts for the conformational flexibility of the PET bulk, based on the six simulations of up to 2 μs conducted for each enzyme.

#### 2.1.2 Negative Charges on the Enzyme Surface Impede, While Apolar and Polar Residues Favor PET Adsorption

LCC with a net charge of +5 and PES-H1 with −5 exhibit contrasting electrostatic potential surfaces (EPS, Figure 2B). While LCC features predominantly neutral and positive EPS areas, PES-H1 showcases extensive negative regions that overlap with the areas of limited PET contacts. This indicates that the presence of negative charges hinders PET binding to the enzyme surfaces, suggesting the potential to deter distal PET interactions by introducing negatively charged residues there. This idea is consistent with the results of Nakamura et al., who demonstrated accelerated binding to PET surfaces with higher positive charge density at the enzyme surface of PET2.^19^ Sagong et al. also showed that modulation of the surface charge affects the PET degradation activity, in their case of *Rhizobacter gummiphilus* PETase (*Rg*PETase).^54^ In particular the introduction of glutamate at distal surface-exposed positions resulted in elevated PET degradation activity of *Rg*PETase. Thomsen et al. observed that, unlike other enzymes such as LCC, PES-H1 creates craters on the PET surface. They attributed this phenomenon to the distal negative surface patch in PES-H1, which they believe impacts the enzyme’s positioning and processing ability after initial contact due to repulsion with the negatively charged PET termini.^17^ From this, we conclude that modulating the surface charge of PET hydrolases provides a possibility to improve enzymatic activity, which we further analyze here by identifying the amino acids most preferred by PET. To this end, we computed the interaction energy *E*_int_ between PET and each amino acid type in all six simulations for both enzymes. We then scaled these energies by the number of interacting residues of each amino acid type, which takes into account the different exposure of the different amino acids, and calculated the average (Figure S2). In Figure 2C, we grouped these *E*_int_ values based on amino acids with similar physicochemical properties. The first observation is that the overall interaction with PET is stronger for LCC than for PES-H1. Moreover, the two enzymes feature distinct preferential binding characteristics. Polar residues dominate LCC interactions, while apolar residues facilitate stronger PET binding in PES-H1. Charged amino acids contribute the least to PET binding for both enzymes. Aromatic residues are almost as important for LCC binding to PET as polar interactions, while they are less relevant for PES-H1 binding. The preference for apolar and also aromatic residues in LCC and the avoidance of charged residues by PET is understandable if one considers the EPS of the PET bulk, which has a predominantly hydrophobic surface in the convex and exposed areas, while stronger positive or negative potentials are only found in concave and buried areas (Figure S3).

#### 2.1.3 Distal Introduction of Alanine and Negative Charge Reduces Local Interaction with PET

Considering the progression from distal to proximal PET binding observed here for PES-H1 but not for LCC, in combination with the avoidance of negative patches on the PES-H1 surface, we speculated that adjusting the surface of LCC to align with PES-H1 might potentially enhance proximal PET binding for LCC and, consequently, impact its enzymatic activity. Such a surface-oriented design strategy gains support from previous studies that revealed that enhanced activity of PET hydrolases is associated with increased enzyme hydrophobicity.^21−23^ To test our assumption, we generated three *in silico* variants per enzyme, each containing three mutations. The mutations were incorporated into the highly active variants LCC F243I/Y127G (IG) (from LCC^ICCG^^3^) and PES-H1 L92F/Q94Y (FY),^12^ which replace the WT enzymes investigated up to this point. The reason for using LCC^IG^ instead of LCC^ICCG^ in this part of the study is that one of the engineered cysteine residues in the latter (D238C) replaced a negatively charged residue, altering the surface charge of the enzyme. This change could complicate the assessment of the impact of the mutations introduced in this study. Before proceeding with the mutations, we confirmed that the variants LCC^ICCG^ (studied below), LCC^IG^ and PES-H1^FY^ behave in MD simulations in the same way as the corresponding WT enzymes (Figure S4).

The mutations we selected mainly involve polar and positively charged residues, which we replaced by apolar and negatively charged residues of similar size. The aim is to increase the overall mobility on the PET surface so that proximal binding becomes more likely. To induce a strong local effect, three mutation sites in close proximity to each other were selected, where preference was given to residues that were in contact with PET in distal areas for at least 75% in one or more of our initial simulations (Figure 2A). For each enzyme, two triple mutants were designed to change the *E*_int_ ratio of polar and apolar residues (LCC^IG^: L66A/S67A/S69A, T211V/S216A/N239L; PES-H1^FY^: T11V/S13A/S14A, Q26L/T27V/T28V) and a third triple mutant was created with the aim to specifically influence the enzyme’s EPS (LCC^IG^: R143E/S145A/R151E; PES-H1^FY^: S13A/R19E/R75E). The LCC^IG^ T211V/S216A/N239L mutations are the only ones located near the active site, since we have observed a dominant PET residency in this region, which may impede PET progression from this site toward the binding cleft. For each of these triple mutants, we performed 100 ns MD simulations in triplicate, starting from the snapshot of the previous simulations where wild-type LCC or PES-H1 had the lowest distance between the PET bulk and the three mutation sites. The mutations effectively altered both *E*_int_ and the EPS (Figure S5), leading to a reduced interaction between the enzymes and PET, as indicated by the less negative *E*_int_ at the mutation sites. The most significant *E*_int_ changes occurred for the LCC^IG^ variants, which is consistent with our design strategy of making the LCC surface more similar to that of PES-H1 to facilitate the reorientation of the enzyme on the PET surface. The introduction of negatively charged residues effectively shifted the EPS in the altered region from positive or neutral to negative, leading to a notable rise in *E*_int_ for the respective LCC^IG^ mutations (R143E, R151E). The impact was less pronounced for the PES-H1^FY^ R19E mutation, as the original PES-H1^FY^ surface was inherently negative, mitigating the mutation’s effect. The implementation of apolar residues, especially alanine, at the expense of polar residues also significantly increased *E*_int_ (*e.g.*, S145A in LCC^IG^, S13A in PES-H1^FY^), while larger apolar residues such as valine or leucine yielded no significant changes or even made the interaction at these sites more favorable (*e.g.*, T211V and N239L in LCC^IG^; Q26L and T28V in PES-H1^FY^). In conclusion, local PET binding was successfully reduced in our simulations by introducing small apolar alanine or negatively charged residues. Therefore, we proceeded with testing the effects of the most promising *in silico* mutations (Figure 2E) on enzymatic activity.

#### 2.1.4 Distal Mutations Have a Major Influence on PET Degradation Activity

**Figure 3:**
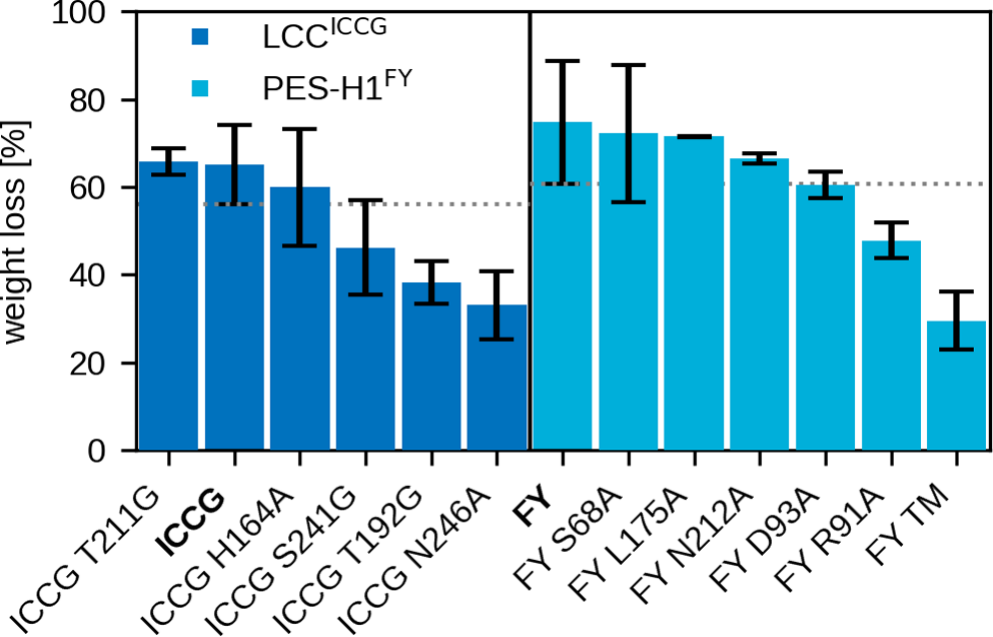
Results from the *in vitro* testing of the original and variants of LCC^ICCG^ (ICCG, left) and PES-H1^FY^ (FY, right). There are five variants per enzyme, which are single-point mutations indicated as axis labels, as well as the PES-H1^FY^ triple mutant (TM) T11 V/S13A/S14A. Variants are ordered according to the percentage weight loss of PET films upon degradation, which serves as a measure for the PET degradation activity. Depolymerization of 60 mg PET film was conducted for 24 h at 70 °C in 1 M potassium phosphate buffer (pH 8) with 60 μg of respective purified enzyme variants. Reactions were performed in duplicate for PES-H1^FY^ L175A and LCC^ICCG^ T211G and for all others at least in triplicate.

To support the calculations, *in vitro* experiments with the triple mutants PES-H1^FY^ T11 V/S13A/S14A with increased hydrophobicity and LCC^ICCG^ R143E/S145A/R151E with increased negative charge were perfromed. PES-H1^FY^ and LCC^ICCG^ were used here as reference enzymes, with the latter replacing LCC^IG^ to allow a more effective comparison with published results.^3,12^ The PET degradation activity was determined with amorphous PET films under specific reaction conditions favored for each enzyme^12^ (see the SI for detailed methods). For PES-H1^FY^, 1 M potassium phosphate buffer has ensured the most efficient hydrolysis rates, which was attributed to its stabilizing effect.^12,55,56^ For an optimal comparison with PES-H1^FY^, LCC^ICCG^, which is not as salt-dependent as PES-H1^FY^,^3^ was also investigated in 1 M reaction buffer. Unfortunately, the LCC^ICCG^ triple mutant could not be expressed, possibly due to the altered surface charge, which could introduce repulsive forces that interfere with native folding. The percentage weight loss of the PET films after 24 h was determined to evaluate the enzymatic PET degrading activity of the PES-H1 triple mutant. As shown in Figure 3 the substitution of three residues in PES-H1^FY^ far away from the binding site led to greatly reduced activity. From this observation, we conclude that a further reduction in the interactions between PET and PES-H1, as shown *in silico* (Figure 2D), and even if it occurs at a distal site, has a diminishing effect on the PET-degrading activity of this enzyme. Since the mutation sites T11 V/S13A/S14A are far away from the active site of PES-H1^FY^, we can exclude a direct impairment of the hydrolysis reaction by these mutations. Instead, our results indicate that when the interactions between the PET hydrolase and the PET material become too weak, enzymatic activity declines. This finding aligns with the Sabatier principle, which posits that optimal catalysis occurs when the interactions between catalyst and substrate are of intermediate strength. Thus, we conclude that modifying the enzyme surface has the potential to enhance PET-degrading activity, warranting further investigation in future studies.

### 2.2 Entry of PET into the Binding Site

#### 2.2.1 Energy Barrier Associated with PET Entry Can Be Overcome by Enhanced Sampling, Leading to Productive Binding Poses

**Figure 4:**
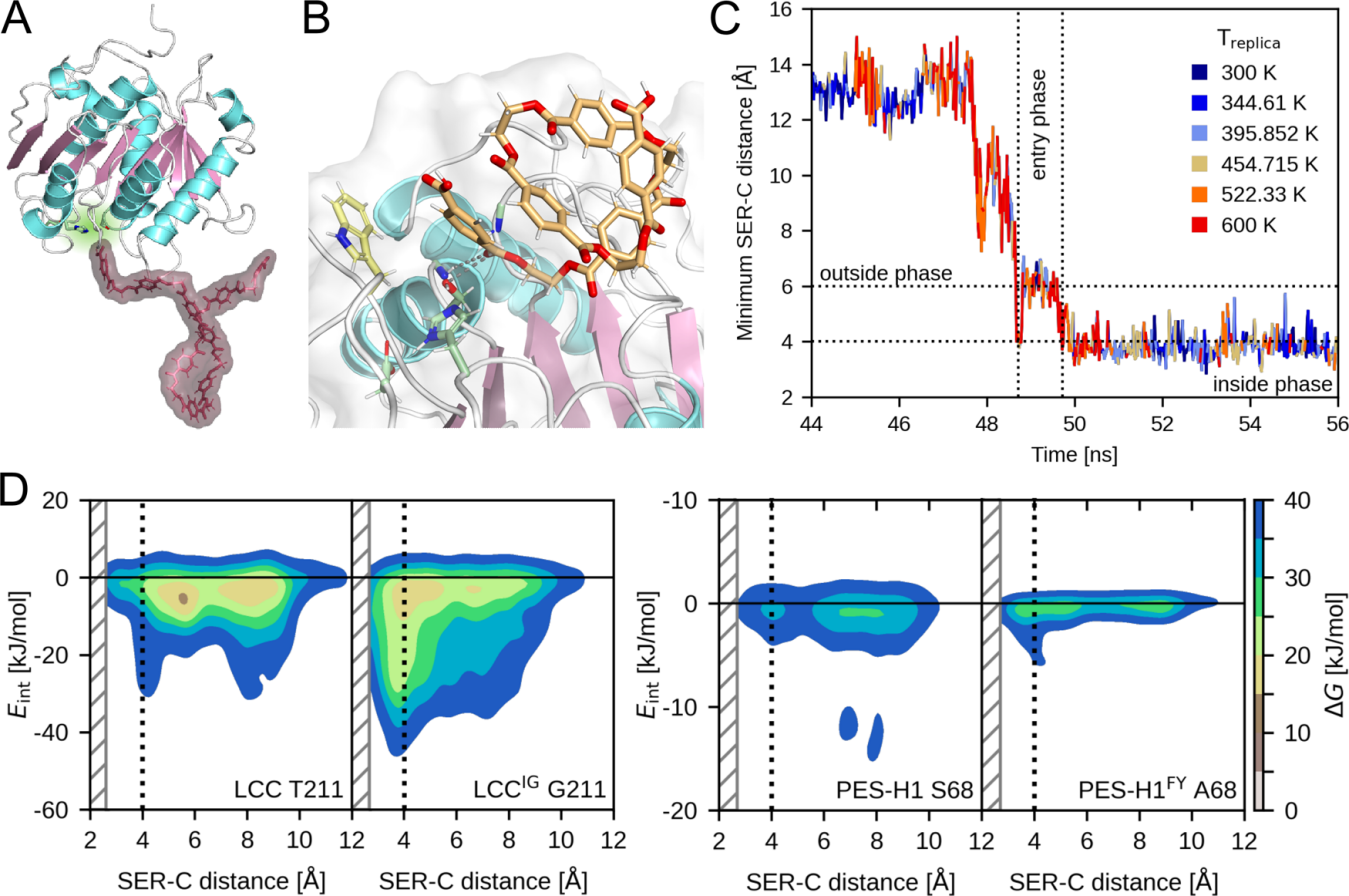
Setup for HREMD simulations and PET-5mer entry results. (A) An exemplary HREMD starting structure is shown to indicate the initial distance between the PET-9mer (shown in red) and the enzyme (active site highlighted in green). (B) PET-5mer entry into the active site was observed, as demonstrated here with a representative snapshot. (C) The entry of the PET-5mer to the active site was monitored by the evolution of the SER–C distance. Here, a representative entry is shown for LCC. The entry was facilitated by the replicas at high energies/temperatures (colored in orange to red), while conformations from replicas simulated at low energies/temperatures are colored in shades of blue (see color key for exact values). The dotted lines indicate the boundaries for the outside, entry, and inside phases, which are defined by the distance criteria (horizontal lines) and resulting times (vertical lines). (D) Free energy surfaces of PET–residue interactions over the SER–C distance obtained for LCC (left) and PES-H1 (right) variants. Results are shown for residue 211 of LCC and residue 68 of PES-H1. The left column shows the results for the corresponding wild-type enzyme and the right column after mutation of the affected residue in LCC^IG^ and PES-H1^FY^. The area for SER–C distance <4 Å harbors productive poses. The gray hatched area indicates distances that were not sampled as the ester carbon atom would be too close to the catalytic serine. The free energies, Δ*G* are given in kJ/mol according to the color scale on the right.

Since the entry of PET into the binding cleft was not observed with conventional MD simulations, our hypothesis was that a considerable energy barrier must be overcome for this to occur. Therefore, we performed HREMD simulations with six replicas per simulation to increase the sampling of the conformational space of PET. For the starting structures of these HREMD simulations, we extracted three conformations from the previous 2 μs MD simulations, applying the selection criterion of a distance between the catalytic serine and PET of at most 5 Å. In addition, we retained only the PET-9mer from the bulk nearest to the enzyme and removed four units from it, leaving a PET-5mer closest to the catalytic serine (Figure 4A). The three starting structures for both enzymes were submitted to HREMD simulations with 115 ns per replica. Some of these HREMD simulations were repeated for statistical reasons, yielding five HREMD simulations for LCC and four for PES-H1 (Table S1). These HREMD simulations enabled to observe several entry events of the PET-5mer into the active site and how this led to the formation of productive binding states (Figure 4B), which we distinguish into *si*- and *re*-face binding of the catalytic serine with respect to the PET ester bond in the active site (Figure S6).^57^ A productive state is present when the distances between the carbon of the nearest PET-5mer ester and the γ-oxygen of the catalytic serine (SER–C distance) and between the carbonyl oxygen of the same ester and the two amino hydrogens of the oxyanion hole scaffold are sufficiently small to allow hydrolysis. As distance cutoff we defined ≤4 Å here.

An example of the PET-5mer entry into the binding cleft is shown in Figure 4C. Here, 12 ns of one of the HREMD simulations of LCC can be seen, where the SER–C distance is initially above 6 Å (the outside phase) and drops to below 4 Å (inside phase) within ≈2 ns (entry phase). Importantly, entry is enabled by exchanging low-energy replicas (corresponding to low temperatures, shown in blue) for high energy replicas (corresponding to high-temperatures, shown in red) immediately before and during entry. Once the entry is complete, the system remains in low-energy replicas. This observation explains why PET entry did not occur in the earlier conventional MD simulations and confirms our assumption that this process involves a significant energy barrier that must be overcome. This was validated by additional conventional 115 ns MD simulations that we run, using the same starting structures as for the HREMD simulations. No productive PET state was observed when simulated at 303 K, but the MD simulations conducted at 343 K, an optimal temperature for enzymatic PET hydrolysis,^4,58^ successfully surpassed the energy barrier for PET entry, yielding both *si*- and *re*-face binding for the PET-5mer for PES-H1 and LCC. Finally, one LCC simulation at 343 K encompassing the entire PET bulk also resulted in *si*-face binding.

To uncover the origin of this energy barrier, we carried out additional analyses and established distinct criteria for pinpointing productive PET-5mer entries (see section 1.8 of the SI), resulting in 29 productive entries for LCC and 15 for PES-H1, accounting for 4.4 and 6.5% of the corresponding MD frames, respectively. Among these entries, 13 and 7 yielded *si*-face binding of the catalytic serine relative to the ester group for LCC and PES-H1, respectively, while the remaining entries resulted in *re*-face binding. The *re*-face binding was previously identified via docking studies,^3^ but quantum mechanics/molecular mechanics studies later revealed that the *si*-face binding is energetically more favorable for hydrolysis^57,59^ and that robust binding of a state unfavorable for hydrolysis could inhibit PET degradation activity.^60^ Thus, although several *re*-face attacks were sampled in our study, hydrolysis is expected to occur mainly via *si*-face attacks. Our analysis of the PET-5mer binding events further shows that the two units that share the ester bond to be hydrolyzed are tightly bound to the enzyme, which is required for the reaction to proceed, while the other PET units interact rather loosely with the enzyme and remain flexible, as evidenced by the various snapshots in Figures S6–S8. This observation contrasts with previous assumptions of a lengthy binding cleft capable of tight binding to multiple PET units simultaneously.^42,61^

From the analysis thus far, it can be concluded that PET has to overcome an energy barrier for entry into the active site to adopt productive conformations. This energy barrier could be driven by conformational changes of PET to fit into the binding cleft. This is supported by recent studies suggesting that a “wrapped” PET conformation is energetically favored in solution, while a “W-shaped” extended conformation, which is crucial for productive binding poses, is observed in association with the enzyme at elevated temperatures.^62^ Such conformational changes, and thus the associated energy barrier, could be influenced by PET–PET and residue–PET interactions, which is investigated next by analyzing intramolecular PET interactions and intermolecular PET–enzyme interactions during productive PET entries.

#### 2.2.2 Intramolecular PET Interactions Hinder Necessary Conformational Changes for Entry into the Binding Cleft

For successful PET binding in the active site of the enzyme, a polymer chain or a part of it has to detach from the amorphous or crystalline PET bulk, which means that nonbonded interactions such as hydrogen bonds and π-interactions within the same chain and with other PET chains must be resolved, in addition to meeting sterical requirements. To assess the impact of the intramolecular PET-5mer interactions on the energy barrier during entry to the binding cleft, we calculated *E*_int_ between PET units during productive entries. In several instances, we observed robust interactions between multiple PET units prior to entry, suggesting an inhibitory effect (Figure S7A). Conversely, individual energy peaks corresponding to favorable intramolecular interactions were detected shortly before or during entry, which probably facilitate the necessary structural rearrangements and interrupt the inhibitory interactions (Figure S7B). In addition, in some cases, entry-promoting effects were found due to newly formed interactions between two units during and/or after entry (Figure S7C).

To more comprehensively assess the predominant interactions present in all simulations of both LCC and PES-H1, as opposed to focusing on individual entry events, we concatenated the HREMD data using all replicas, with the higher replicas weighted, for either enzyme and calculated two-dimensional (2D) free energy surfaces (FESs) (Figure S9). One coordinate in these FESs are the interaction energies between two PET units that are not directly adjacent, which allows us to monitor the intramolecular PET-5mer interactions. To correlate this with the PET-5mer entry, we used the SER–C distance as a second coordinate and distinguish between productive and nonproductive regions with a cutoff value of 4 Å. All FESs indicate that nonproductive PET-5mer states and also those during entry with SER–C distances below 6 Å but above 4 Å are energetically stabilized, which suggests that PET–PET interactions hinder rather than promote PET-5mer entry into the active site.

In our opinion, the most promising approach to overcome this inhibitory effect is to improve the preprocessing of PET waste. Tarazona et al. studied the surface erosion process of PET to differentiate between the PET surface and the bulk properties in PET degradation.^63^ They found that enlarging the PET surface, *e.g.*, by using micronized PET particles, increasing the surface amorphization and lowering the surface glass transition temperature due to the plasticization effect of water promotes the degradation activity by increasing the PET accessibility and mobility to overcome intramolecular constraints. This is also consistent with a recent study by Schubert et al., in which chain mobility and the number of available sites for endotype chain scission, which depend on the degree of crystallinity, are postulated as the activity-limiting factor causing the initial lag phase for the release of the soluble product.^7^ Another biotechnological approach to mitigate this hindering effect could be to design enzymes to introduce residues that enhance enzyme–PET interactions, which would promote and counterbalance the loss of PET–PET interactions upon entry into the binding cleft.

#### 2.2.3 PET–Enzyme Interactions Can Also Hinder PET Entry

To assess the contribution of enzyme–PET interactions to the energy barrier during PET entry, we determined the intermolecular interactions between the PET-5mer and enzyme. To this end, we first examined individual entry histories and identified effects that promote or inhibit entry by calculating *E*_int_ between the PET-5mer and residues within 3 Å during entry. PET-5mer entry can be hindered by strong PET–enzyme interactions prior to entry or by repulsion of the PET-5mer within the binding cleft, so that PET-5mer states outside the cleft are favored (Figure S8A). Conversely, an entry-promoting effect has favorable interactions during entry, which facilitate structural PET-5mer rearrangements that overcome PET–PET interactions (Figure S8B). Furthermore, PET-5mer entry gets supported through stabilizing interactions for productive PET5-mer poses, which compensate for the loss of PET–PET interactions (Figure S8C).

To obtain a more general overview, FESs were generated using a similar approach as before, but this time for the PET–enzyme interactions. The SER–C distance was again used to monitor the entry process, while *E*_int_ between the PET-5mer and selected residues was used as a second coordinate. The selection and mutation of specific residues allows us to investigate their effects on the corresponding FES. To gain insight, we first examined the activity-enhancing mutations in LCC^IG^ and PES-H1^FY^ using HREMD simulations. The main conclusions from the results summarized in Figure S10 are that some of these mutations (*e.g.*, F243I in LCC) stabilize productive states, which can be inferred from the appearance of new free energy minima at SER–C distances below 4 Å and less pronounced minima for distances above 6 Å after the mutation, suggesting weaker interactions with the PET-5mer in the nonproductive states. This is the desired behavior after mutation, in addition to a smooth transition from the nonproductive to the productive region by avoiding energy barriers in between. In some instances, however, the mutation of a specific residue results in only a slight advantage for the entry of the PET-5mer, as it eliminates solely unproductive energy minima without notably stabilizing the productive state (*e.g.*, for Y127G in LCC). The least desirable effect is that it appears to be more difficult for the PET-5mer to enter the binding site after the mutation, as there are increased interactions with the PET-5mer in nonproductive states, corresponding to pronounced minima of free energy in this region (*e.g.*, for L92F in PES-H1).

Considering this, we explored the impact of nearby active site residues that interact with PET (Figure S11). We aimed to identify those without entry-promoting FES attributes and mutate them to enhance PET entry. The identified residues were substituted by glycine or alanine, with larger amino acids deliberately omitted to prevent steric hindrances. The lone exception is the PES-H1^FY^ H184S variant, positioned adjacent to a conserved tryptophan (PES-H1: W155, LCC: W190), which may enhance catalytic activity due to its “wobbling”.^41^ The mutation of H184 to serine was anticipated to enhance the flexibility of the nearby tryptophan due to reduced steric demands. This resulted in a total of 14 mutation sites that were evaluated for each enzyme. For each mutant, an HREMD simulation was performed (see Table S1 for the list of HREMD simulations) and the FES calculated for the original and mutated residue. In many cases, the overall goal of the *in silico* mutations to facilitate PET-5mer entry into the active site was achieved. For these cases the FES is shown in Figure S12, and they include mutations with improved stabilization of productive states (PES-H1^FY^: S68A, D93A; LCC^IG^: T211G, N246A), reduced interaction in nonproductive states (PES-H1^FY^: S68A, R91A, D93A, N212A; LCC^IG^: T192G, S241G), or smoother transitions toward productive states without intermediate states where the PET-5mer could get trapped (PES-H1^FY^: S68A, D93A; LCC^IG^: HIS164A, T211G, S241G, N246A). The PES-H1^FY^ H184S variant could neither improve the interactions at position 184 nor the interactions of the “wobbling” tryptophan with the PET-5mer. The five most promising *in silico* mutations per enzyme were then evaluated experimentally, using the same protocol as employed for the previous PES-H1^FY^ triple mutant to determine the PET degradation activity.

#### 2.2.4 Kinetic Experiments Reveal an Increase in Activity for the PES-H1^FY^ S68A Mutation

In order to link simulation and experiment, the activity of the mentioned mutations introduced in the PES-H1^FY^ and LCC^ICCG^ enzymes was determined. The percentage weight loss of the PET films shown in Figure 3 indicates that none of the variants resulted in a significant increase in the degradation activity. PES-H1^FY^ R91A, LCC^ICCG^ T192G, and LCC^ICCG^ N246A showed significantly lower degradation activity than PES-H1^FY^ and LCC^ICCG^, respectively. Interestingly, these are also the three variants with the least promising FES profiles among the ten selected variants (Figure S12). The FES improvements in these mutants were mainly related to the destabilization of the nonproductive states, while the productive state did not (considerably) gain in stability. In addition, there is also the possibility that other factors may influence the experimental outcome and thus mask improvements due to mutations. It should also be mentioned that the reproducibility of the experiments was problematic despite uniform experimental conditions in all experiments, which we suggest can be attributed to influences of the inherent properties of the PET films such as crystallinity or molecular weight distribution.^14^

To reduce potential masking effects and identify subtle effects on individual steps in PET degradation, we analyzed the degradation kinetics using a previously described turbidimetric assay using PET nanoparticles (PET-NP) that measures turbidity changes at 600 nm over time.^12,64^ Here, not only the substrate load [*S*_0_] was varied while maintaining a fixed enzyme concentration [*E*_0_], as done in conventional Michaelis–Menten kinetics measurements (^conv^MM), but also the reverse approach with varying enzyme concentration and fixed substrate load was followed, which is called inverse Michaelis–Menten (^inv^MM) and is a commonly used approach for kinetic measurements with insoluble substrates such as PET.^16,25,65−71^ To further ensure a more homogeneous distribution of the substrate, PET-NP were used instead of a PET film, which should lead to more reproducible results.^71^ This approach was used to evaluate the three most interesting candidates per enzyme that showed the most improvement in their FES profiles upon mutation while exhibiting similar PET degradation activity to PES-H1^FY^ and LCC^ICCG^, respectively, namely PES-H1^FY^ S68A, D93A, N212A and LCC^ICCG^ H164A, T211G, S241G (Tables 1 and S3).

**Table 1:** Michaelis–Menten Constants *K*_m_ Were Derived from a Nonlinear Fit to Experimental Data for ^conv^MM and ^inv^MM of Both Enzymes, LCC^ICCG^ (ICCG) and PES-H1^FY^ (FY), and Variants Thereof^a^

	^conv^*K*_m_ [g/L]	^inv^*K*_m_ [μm]	*k*_cat_ [1/min·μM]	^molar^*K*_m_ [μM]	^molar^η [1/min·μM^2^]
LCC^ICCG^	0.215 ± 0.059	0.365 ± 0.061	0.047	0.357	0.132
ICCG H164A	0.095 ± 0.063	0.861 ± 0.164	0.032	0.278	0.114
ICCG T211G	0.091 ± 0.068	0.430 ± 0.130	0.042	0.223	0.189
ICCG S241G	0.250 ± 0.068	0.234 ± 0.070	0.054	0.357	0.151
PES-H1^FY^	0.391 ± 0.109	0.892 ± 0.105	0.088	1.036	0.085
FY S68A	0.357 ± 0.117	0.278 ± 0.053	0.084	0.728	0.115
FY D93A	0.501 ± 0.180	2.017 ± 0.481	0.040	2.434	0.016
FY N212A	0.481 ± 0.174	1.767 ± 0.318	0.068	1.564	0.043

a The secondary parameters *k*_cat_, ^molar^*K*_m_, and ^molar^η are also provided.

Improved ^inv^*K*_m_ values were attained for some of the variants compared to the parent enzymes. In particular, an approximate 3-fold decrease in ^inv^*K*_m_ was detected for the PES-H1^FY^ S68A variant compared to PES-H1^FY^, which indicates a higher enzyme–substrate affinity for PES-H1^FY^ S68A. For the other two PES-H1^FY^ variants, higher ^inv^*K*_m_ values than for PES-H1^FY^ suggest a decreased affinity, which is supported by similar trends in the ^molar^*K*_m_ values. For LCC^ICCG^ the results are less clear. The higher ^inv^*K*_m_ values for LCC^ICCG^ H164A and T211G than for LCC^ICCG^ are not mirrored by the corresponding ^molar^*K*_m_ results, which are lower, while the turnover rate constant *k*_cat_ could also not be improved. For the LCC^ICCG^ S241G variant, a reduction in ^inv^*K*_m_, a similar ^molar^*K*_m_ value as for LCC^ICCG^, and an increase in *k*_cat_ were observed, but these improvements are not statistically significant. Combining affinity and turnover rate into ^molar^η = *k*_cat_/^molar^*K*_m_ yields the molar catalytic efficiency. First of all, it should be noted that a higher catalytic efficiency was observed for all LCC^ICCG^ variants than for the PES-H1^FY^ variants. A comparison of these figures per enzyme indicates that PES-H1^FY^ S68A and LCC^ICCG^ T211G outperform PES-H1^FY^ and LCC^ICCG^, respectively, while LCC^ICCG^ S241G shows a slight improvement. Thus, the most promising mutations revealed by experiments are also the mutations with the most promising FES changes identified by our simulations (Figure 4D). In particular for the T211G mutation in LCC^ICCG^ we succeeded in creating a smooth free energy path leading to the productive state, which corresponds to the largest increase in ^molar^η with respect to the parent enzyme for all variants studied. With the S68A mutation in PES-H1^FY^ we were able to abolish the stabilization of unproductive states, while the productive state gained stability.

In conclusion, we characterized PET entry features by comparing the FES of residues in parent enzymes with mutations, linking FES profiles to experimental enzymatic activity. This analysis supported the notion that altering the FES to facilitate PET entry by eliminating energy barriers and stabilizing the productive state was best exemplified in PES-H1^FY^ S68A and LCC^ICCG^ T211G, resulting in enhanced catalytic activity. Our findings further suggest improved enzyme efficiency particularly at low enzyme concentrations, which is advantageous for high substrate concentration scenarios in industrial applications where maintaining low enzyme levels is essential due to cost or feasibility constraints.

#### 2.2.5 Principal Component Analysis Reveals That PET Can Enter via Three Pathways

To complete our simulation analysis of PET entry into the binding cleft, we next present the complete pathway of how the PET-5mer reaches its final productive state in the active site. To this end, we compiled data from all entry events across all variants simulated for LCC and PES-H1, respectively, creating robust data sets for principal component analysis (PCA) of PET-5mer ester carbon distances to C_α_ atoms within a 10 Å range during entry. In doing so, we do not limit our analysis to the assumption that there can only be one such pathway, but aim to identify all possible pathways. By analyzing the resulting principal components (PCs) for free energy (Δ*G*) along the first two PCs (Figure S13A,B), we identified distinct productive and nonproductive states in the FESs, indicating clear separation and localization of productive states within Δ*G* minima regions. Furthermore, examining the contributions of amino acids crucial for PET-5mer entry to PC1 revealed 49 significant positions, underscoring the complexity of the entry pathway and validating the suitability of HREMD for unbiased sampling of PET conformational space.

**Figure 5:**
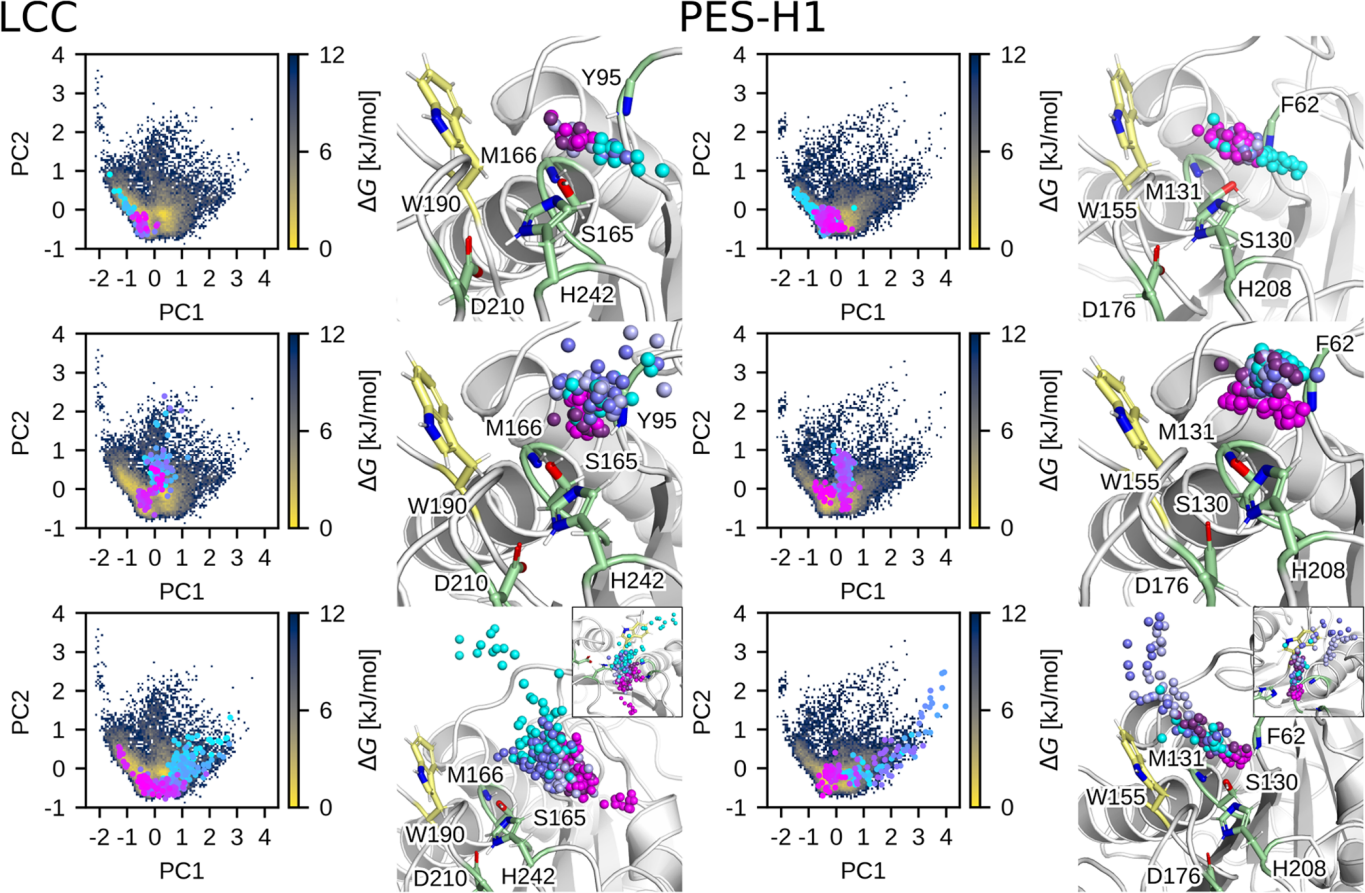
Three preferred entry pathways of the PET-5mer as revealed by PCA applied to the HREMD data involving the simulations of all LCC (left) and PES-H1 (right) variants performed in this work. For each pathway, a representative example is shown. The pathways are mapped as circles on the FES in the PC1, PC2 plane of either enzyme and are also shown (as spheres) together with the corresponding enzyme structure. The progress of each pathway is highlighted by the color change from cyan to magenta. In the structure plots, the spheres show the positions of the PET-5mer ester carbons that are finally productively bound.

The FES along the enzyme–PET contact PCs further indicate that the PET-5mer uses not a single exclusive pathway, but rather three preferred entry routes in both enzymes. A representative trajectory for each pathway is mapped onto the FES and enzyme structures (Figure 5), illustrating the PET ester carbon’s progression toward its productive pose through a color transition from cyan to magenta. The first pathway leads from the side opposite the “wobbling” tryptophan to the active site (Figure 5, top). The second pathway goes directly from the exterior to the catalytic serine (Figure 5, middle), while in the third pathway, the ester bond slides over the benzene ring of the “wobbling” tryptophan until it reaches its final position for hydrolysis (Figure 5, bottom). Visual inspection of a number of entry pathways revealed a significant flexibility of the ester carbon position, involving multiple reorientations of the ester position, even when close to the active site, until a final productive conformation is attained. Both enzymes exhibit the same entry pathways, with the first route being the most clearly defined and energetically favored in both cases. As for the second-preferred route, the LCC shows a preference for the direct entry of PET-5mer as found in the second pathway, which is also better defined than in PES-H1, while in the latter these arguments apply to the third pathway.

The preference for a specific entry pathway is impacted by interactions with particular residues, which is mainly reflected by their contribution to the dominant principal component (Figure S13C). The residues influencing the negative PC1 range shape the first pathway positioned at negative PC1 values, while the residues linked to the positive PC1 range affect the third pathway positioned at positive PC1 values. The magnitude of the residues’ contributions to PC1 mirror their impact on each pathway. The second pathway aligns with PC1 ≈ 0 and thus lacks substantial contributions from residues along PC1. The positions mutated in PES-H1^FY^ and LCC^ICCG^ correspond to residues influencing PC1: residues 92 and 94 in PES-H1 contribute negatively to PC1, influencing the primary pathway, and residue 127 in LCC has a similar effect. The other mutated residue at position 243 in LCC^ICCG^ contributes positively to PC1, influencing the third pathway. For several of the positions that we mutated in the current study, we also find a strong contribution to PC1. In particular, position 68 of PES-H1 makes the largest contribution to PC1, which turned out to be the PES-H1^FY^ variant with the highest catalytic efficiency after its mutation from serine to alanine (Table 1). Position 68, alongside position 209 in PES-H1 (corresponding to position 243 in LCC and essential for LCC^ICCG^’s activity), plays a significant role at one end of the catalytic binding cleft, potentially obstructing the predominant PET entry pathway (Figure S11). To test this theory, additional *in silico* PES-H1 variants were developed for position 68 (S68I, S68F, S68Q, S68D, S68K), and HREMD simulations were conducted for each variant to analyze PET-5mer entry using the previous FES method. Despite investigating various side chain properties at position 68, results from Figure S14 suggest that the initial strategy of mutating PES-H1^FY^ S68 to alanine was advantageous. Mutating this residue to other amino acids led to stabilization of nonproductive states and isolated regions in the FES, indicating increased difficulty transitioning of PET to the productive state. Hence, interactions involving this residue are commonly unhelpful for PET entry, underscoring the efficacy of steric hindrance removal as achieved in the PES-H1^FY^ S68A mutation.

In summary, the PET entry process exhibits a certain degree of variability due to the considerable flexibility of the PET chain and persistent PET–PET interactions during entry as shown above, but also due to the possibility that the ester bond that is eventually cleaved can reach the active site by three major routes. Nonetheless, for both PES-H1 and LCC we have identified one pathway as the predominant one, which is identical for both enzymes and is consistent with the findings by Falkenstein et al., who proposed this pathway as the PET entry pathway.^48^ However, in view of the highly dynamic situation during PET insertion observed here, the proposal of stepwise PET degradation by “hopping and sliding” and successive cleavage of PET ester bonds as the primary degradation mechanism is less tenable.

## 3 Conclusions

In this study, we systematically investigated the binding process of PET to LCC and PES-H1 to reveal the entire pathway from initial adsorption to a PET bulk to the entry of a single PET chain into the binding site, using an approach encompassing simulations and experiments as shown in Figure S1. Our main observation regarding PET surface binding is that decreasing adsorption affinity, achieved by substituting polar residues predominantly with alanine on the PES-H1 surface far away from the active site, significantly diminished enzymatic activity. The suboptimal performance of this PES-H1 surface variant should not be viewed as a regression but rather as an opportunity to explore the impact of modifying surface residues distal from the active site. This study can serve as a catalyst for future research on the interaction between PET hydrolase surfaces and PET material to identify the optimal interaction strength between the two, in accordance with the Sabatier principle, while considering potential protein aggregation and changes on the PET surface during PET degradation. In our study on PET chain entry into the binding site, we observed three distinct pathways rather than a single route. We identified energy barriers from PET–PET interactions and interactions with amino acid residues that may impede PET from assuming a productive state. Analyzing the free energy and principal components of these interactions in wild-type and mutated LCC and PES-H1 enzymes revealed potential energy barriers arising from stabilizing unproductive states. Enhancing the transition from unproductive to productive states by mutations could boost enzymatic activity, which we confirmed by simulations and kinetic experiments with the improved S68A mutant of PES-H1^FY^ and the T211G mutant of LCC^ICCG^. In summary, our pioneering methodology synergized HREMD simulations and free energy analyses to dissect substrate entry pathways and pinpoint barriers and guided mutation strategies for PET hydrolase, offering guidance for future engineering to improve their applicability.

## Supporting Information Available

Additional experimental details, materials, methods; variants studied by HREMD simulations; oligonucleotides used for site directed mutagenesis; examples of *si*- and *re*-face binding of the catalytic serine (here S165 as LCC is shown) with respect to the ester bond of PET binding in the catalytic site; mutation sites and results of the variants addressed using the FES approach (PDF)
